# Reversed cortico-medullary differentiation in kidneys on fetal magnetic resonance imaging — a case series

**DOI:** 10.1007/s00467-025-06938-4

**Published:** 2025-08-21

**Authors:** Monika Bekiesinska-Figatowska, Jaroslaw Madzik, Marcin Ring, Agata Skorka, Marta Smyk, Ewa Obersztyn

**Affiliations:** 1 Department of Diagnostic Imaging, Institute of Mother and Child, Ul. Kasprzaka 17a, Warsaw, 01-211 Poland; 2 Department of Medical Genetics, Children’s Memorial Health Institute, Al. Dzieci Polskich 20, Warsaw, 04-730 Poland; 3 Department of Medical Genetics, Institute of Mother and Child, Ul. Kasprzaka 17a, Warsaw, 01-211 Poland

**Keywords:** Fetus, Kidneys, Magnetic resonance imaging (MRI), Reversed signal intensity (SI)

## Abstract

The reversed cortico‑medullary differentiation in fetal kidneys on ultrasound has been described in the literature, but there have been no descriptions of such a finding on fetal magnetic resonance imaging (MRI) so far. The authors present three unrelated fetuses with hyperechoic kidneys on ultrasound (US) and reversed signal intensity of their cortex and pyramids on SSFSE/T2WI and FIESTA images on magnetic resonance imaging (MRI). All of them shared the same deletion of the long arm of chromosome 17 in the 17q12 region, responsible for the expression of clinical features of renal cysts and diabetes (RCAD) syndrome. All of them had multiple tiny kidney cysts on US after birth. This specific finding on fetal MRI may point at this specific genetic condition.

## Introduction

We have been performing fetal magnetic resonance imaging (MRI) since the beginning of this century; however, no earlier than 2020 did we notice the unusual appearance of fetal kidneys with a reversed signal intensity (SI) of cortex and pyramids on SSFSE/T2WI and FIESTA images. To date, we have not found any descriptions of this kind of MR appearance in the available literature. The article by Cassart et al. last year [1] reporting the reversed cortico‑medullary differentiation in fetal kidneys on ultrasound (US) prompted us to revisit our findings, which we present here.

## Case reports

**Fig. 1:**
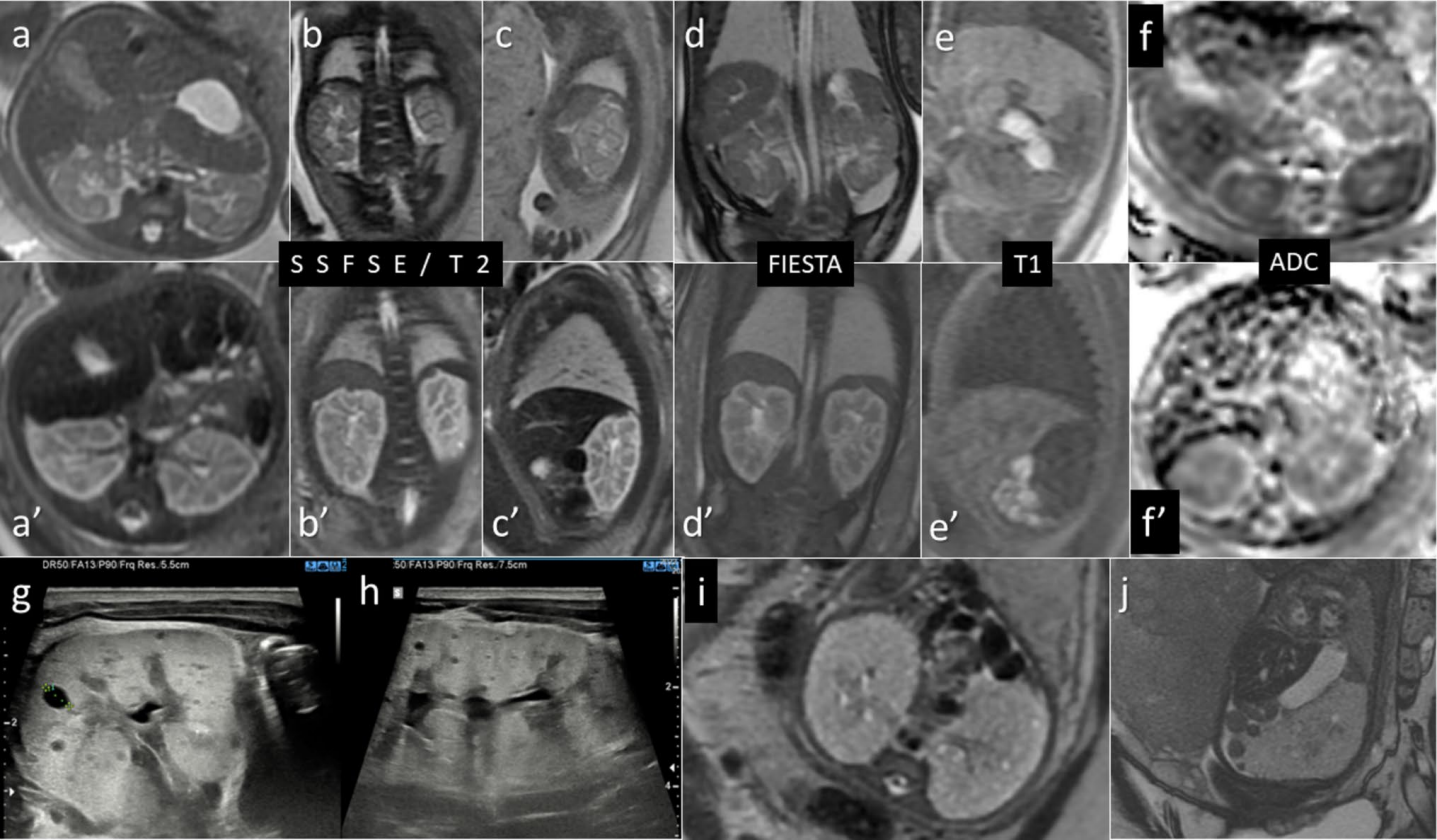
Comparison of the normal SI of the fetal kidneys (**a**–**f**, top row) and of the reversed pattern in case no. 1 (**a'**–**f'**, middle row) in corresponding sequences and sections at the same gestational age of 36 weeks. Postnatal ultrasound of the cystic kidneys in neonate no. 1 at day of life 7 (**g**–**h**, bottom row). Completely different MRI appearance of ARPKD in a fetus for comparison (**i**–**j**, bottom row)

We had three cases of fetal kidneys with T2-hyperintense cortex and T2-hypointense medulla reversal of the correct appearance. In all of them, the kidneys were enlarged and hyperechoic on US of the second or third trimester, and the inverted pattern of cortex and medulla was only found on MRI. All fetuses had enlarged kidneys on MRI performed between 32 and 36 weeks (range 52–58 mm), weaker diffusion restriction in the kidneys than under normal conditions, had their bladders filled, and no oligohydramnios/anhydramnios. Figure 1 presents the MRI findings in fetus no. 1 compared with normal appearance of the fetal kidneys and with postnatal sonographic appearance.

In male case no. 1, amniocentesis was performed due to the first trimester sonographic abnormalities (tricuspid valve regurgitation, regurgitation wave A, enlarged urinary bladder). The array comparative genomic hybridization (aCGH) analysis of the DNA extracted from uncultured amniocytes showed the deletion at 17q12 region ~ 1.4 Mb in size encompassing the region of renal cysts and diabetes (RCAD) syndrome (OMIM 137920, ORPHA: 93111). The deletion included 15 protein-coding genes, including the *HNF1B* gene. So far, no parental examination has been performed to assess the origin of the deletion of 17q12. At the age of 14 months, the boy’s kidney function parameters were within normal limits.

In female case no. 2, there was postnatal appearance of bilateral multicystic kidney, so the *PKHD1* gene was sequenced and no pathogenic variants were identified. As there was also a Müllerian duct anomaly (bicorporeal uterus with double cervix), microdeletion 17q12 syndrome was then suspected. The results of Multiplex Ligation-dependent Probe Amplification (MLPA; Kit P297-D1, MRC-Holland, Amsterdam) revealed heterozygous deletion in the region of the long arm of chromosome 17 including genes *ZNHIT3*, *LHX1*, AATF, *ACACA*, and *HNF1B*, which refers to the region typical for 17q12 microdeletion syndrome. The parents have declined testing themselves. At the age of 4.5 years, the girl has normal kidney function, and on US, the kidneys show increased cortical echogenicity, preserved cortico-medullary differentiation, and a few cortical cysts up to 4 mm. No more detailed examination of the reproductive system was performed.

The same 17q12 deletion as in case no. 1 was identified postnatally in male case no. 3 using the same technique (aCGH analysis of the DNA extracted from blood). The results of MLPA in the healthy young parents are pending. Up until the age of 1 month, the boy showed no biochemical abnormalities, including kidney function parameters.

## Discussion

Starting from the pattern of inverted SI from the kidney cortex and medulla on fetal MRI in three different fetuses, we obtained genetic data of the same abnormality.

From the radiological point of view, it is important that this pattern has been detected only on fetal MRI and not on US. The authors of the article that inspired us saw this abnormality on fetal US, but in other entities [1], while in our three cases on fetal US hyperechoic kidneys were described.

Non-hereditary, hyperechoic fetal kidneys can be found in obstructive dysplasia, bilateral multicystic kidney disease, nephroblastomatosis, renal vein thrombosis, ischemia, infectious and metabolic diseases, nephrotic syndrome, and aneuploidy. Enlarged hyperechoic kidneys without associated malformations are most frequently found in autosomal recessive and dominant polycystic kidney diseases [2].

Renal cysts and diabetes syndrome (RCAD) is caused by anomalies of the gene for hepatocyte nuclear factor 1 beta (*HNF1B*) and is also called maturity-onset diabetes of the young type 5 (MODY5). It seems to be the second most common etiology of cystic kidney diseases in childhood nowadays. The clinical manifestations include kidney anomalies (cysts, hypo-/dysplasia, hypomagnesemia) and pancreatic anomalies (diabetes mellitus, pancreatic atrophy), and can also include abnormalities of the liver and genital tract [3] as in our case no. 2. If kidney cysts are not associated with disturbances in glucose metabolism, the term *HNF1B*-associated kidney disease is used.

Having acquired the knowledge of *HNF1B*-related kidney disease in our unborn patients, we found the related literature with descriptions of fetal US and MRI [4, 5]. However, there is no information about reversed cortico-medullary SI pattern on fetal MRI there. Cleper et al. performed fetal MRI in four out of six cases, but they report just “abnormal signal intensity” of the kidneys. In one fetus, “irregular kidney contour with a hyperintense rim” was reported, and this argues in favor of taking another look at these studies for similarities to our cases. In two out of these four fetuses, cysts were seen already in the prenatal period on MRI (in our material only on postnatal US). In all six fetuses, the kidneys were hyperechoic on prenatal US [4]. Madariaga et al. reported 12 fetuses with *HNF1B* mutation, and in all cases, fetal US was performed and revealed abnormalities from 14 gestational weeks onwards: hyperechoic kidneys in six, cystic changes in seven (in one fetus, one kidney was hyperechoic and the other one was cystic) [5].

In the paper by Cassart et al. the authors wrote that in their cases “in prenatal imaging, complementary … MRI does not appear useful since cortico-medullary differentiation is far better evaluated at US due to its higher spatial resolution” [1]. However, being pediatric radiologists, they performed fetal US by themselves, while in our country — as in many others — fetal US is in the hands of obstetricians. US is and will remain the first-line imaging modality in the diagnostics of fetuses; however, fetal US — as US in general — is strongly dependent on the sonographer’s skills, knowledge and experience. Therefore, fetal MRI is a more objective method. In our cases, this pattern of kidney changes would not have been depicted without MRI. To the best of our knowledge, this is the first report on inverted SI of kidney cortex and medulla on fetal MRI.

In conclusion, our hypothesis is that the reversal of kidney cortico-medullary differentiation on fetal magnetic resonance imaging may point to the specific genetic condition associated with 17q12 deletion syndromes and may distinguish these entities from others, manifesting as hyperechoic kidneys on fetal ultrasound. This requires further studies and referring pregnant women with hyperechoic fetal kidneys to MRI.

## Summary

### What is new

- Reversed kidney cortico-medullary differentiation on fetal MRI points at 17q12 deletion syndrome and RCAD, and may distinguish it from other abnormalities, manifesting as hyperechoic kidneys on fetal ultrasound.

## Data Availability

The data that support the findings of this study are not openly available due to reasons of sensitivity and are available from the corresponding author upon reasonable request.
